# The Effectiveness of Poly-(4-vinyl-N-hexylpyridiniumbromide) as an Antibacterial Implant Coating: An *In Vitro* Study

**DOI:** 10.1155/2011/859140

**Published:** 2011-12-21

**Authors:** L. Ringenberg, A. Winkel, O. Kufelt, P. Behrens, M. Stiesch, W. Heuer

**Affiliations:** ^1^Department of Prosthetic Dentistry and Biomedical Material Science, Hannover Medical School, 30625 Hannover, Germany; ^2^Institute of Inorganic Chemistry, Leibniz University Hannover, 30167 Hannover, Germany

## Abstract

The clinical success of osseointegrated dental implants depends on the strong attachment of the surrounding hard and soft tissues. Bacterial adhesion on implant surfaces can cause inflammatory reactions and may influence healing and long-term success of dental implants. Promising implant coatings should minimize bacterial adhesion, but allow epithelial and connective tissue attachment. Therefore, the present study has examined the bioactive effect of poly-(4-vinyl-N-hexylpyridiniumbromide) regarding typical oral bacteria as well as cytotoxicitiy to human cells considering different methods of connecting polymers to silicate-containing surfaces. The results revealed that the application of putative antibacterial and biocompatible polymer in coating strategies is affected by a variety of parameters. Published findings regarding reduced bacterial adhesion could not be verified using oral pathogens whereas hexylated polymers seem problematic for strong adhesion of soft tissue. Concerning innovative coatings for dental implants basic aspects (surface roughness, thickness, alkylation, combination with other polymers) have to be considered in further investigations.

## 1. Introduction

Oral implants play an important role in restorative dentistry and their clinical success has resulted in their widespread use [1]. The application of osseointegrated dental implants has been shown to be an excellent method for replacing missing teeth in patients for partial or total rehabilitation. Besides esthetic improvements and favourable phonetics, implants facilitate the restoration of mastication. Today, implant-supported prosthetic supraconstructions are of increasing importance and have partially replaced conventional prosthetic treatments [2]. Since the implementation of oral implants forty years ago, several studies have analyzed the improvements in implant material, implant surface, and implant design, in order to achieve optimal osseointegration [3–5]. While there is considerable information and progress on the osseous healing of implants, little is known about the process of bacterial interactions between the implant surface and the surrounding tissue [1]. It is only clear that bacterial adhesion on implant surfaces endangers healing and long-term success of dental implants [3, 6].

Biofilm formation on solid surfaces within oral cavity such as teeth, prostheses, or implant-anchored supraconstructions already begins within minutes after dental hygiene [7, 8]. First, a thin removable layer formed by salivary biopolymers and various proteins appears, called “acquired” or initial pellicle, followed by primary bacterial colonizers, usually aerobic and facultative anaerobic gram-positive coccoids, such as different Streptococcus species (e.g., *S. sanguinis*, *S. salivarius*, *S. mitis*, *S. oralis*) [9, 10]. This initial colonization together with subsequent deposition of protective extracellular matrices creates required preconditions for the successive incorporation of secondary microorganisms, especially anaerobic gram-negative coccoids and rods [9–11]. Depending on the bacterial composition and amount of growing biofilm, inflammatory reactions in the periodontal and peri-implant soft and hard tissues occur, which can lead in worst case to progressive bone resorption and early implant failure [7, 12–15].

Therefore, the development of antibacterial effective coatings especially for oral application is of increasing importance [16]. Towards this goal, Tiller et al. have shown that poly-(4-vinyl-N-alkylpyridiniumbromide) (pVP) coated on glass slides kills more than 90% of deposited *Staphylococcus aureus* cells and 99% of the gram-positive bacterium *Staphylococcus epidermidis* as well as the gram-negative bacteria *Pseudomonas aeruginosa* and *Escherichia coli* when used [16]. However, this study did not include primary colonizers from the oral cavity with pathogenic relevance.

Beside the required antibacterial effect of coating strategies, tight junction of surrounding soft tissue and subsequent long-lasting occlusion is of major importance for the long-term success of oral implantation strategies [17]. As described previously by Heuer et al., gingival fibroblast adhesion on pVP-coated titanium as well as proliferation capacity of cells might be reduced [18]. This decrease in biocompatibility was partially superable by modification of the polymer linkage, demonstrating the importance of different binding strategies for the biological effectiveness of coating substrates [18, 19].

The aim of the present study was to verify the known antibacterial properties of differently coated glass slides with poly-(4-vinyl-N-hexylpyridiniumbromide) regarding typical oral bacteria (*S. mutans*,* S. sanguinis*). In order to share some light on effects of surface enhancement and modification, different procedures of binding the polymer to silicate-containing surfaces such as nanoporous and amorphous silicon dioxide were used. Together with a reasonable biocompatibility, this coating strategy would offer opportunities for future applications on implant ceramics in prosthetic dentistry. 

## 2. Material and Methods

All experiments were based on purified round glass sheets (0.13–0.16 mm in thickness and about 1.13 cm² in surface area). The discs partially served as a substrate for surface modifications applying silica layers in amorphous, respectively, nanoporous conformation. Finally, all samples were coated with the potentially bactericidal polymer poly-(4-vinyl-N-hexylpyridiniumbromide) or an ineffective, nonhexylated polymer serving as a control [16].

### 2.1. Creation of Amorphous and Nanoporous Surfaces

In the present study, different kinds of glass surfaces were used. Beside untreated glass, amorphous and nanoporous structures were coated with bactericidal polymer.

To generate nanoporous glass slides, the structure-regulating agent EO_20_PO_70_EO_20_ (Sigma-Aldrich, Germany) was solved in a mixture of EtOH, H_2_O, and HCl before adding Tetraethoxysilan (TEOS). Glass slides (Menzel, Germany) were spin coated and dried at 60°C overnight. Finally, organic components were removed by calcination at 415°C.

Procedure for amorphous surfaces was comparable with the exception of missing the structure-regulating agent.

### 2.2. Pretreatment of Glass Samples

**Cleaning.**

Uncoated glass slides were cleaned by sonication in Aceton and Ethanol. Amorphous and nanoporous surfaces were not cleaned furthermore.

**Activation.**

All glass samples were treated with piranha etch (H_2_SO_4_ : H_2_O_2_ 6 : 4) for 15 min.

**Coating with APTMS.**

After activation, slides were covered with a 10% 3-Aminopropyltrimethoxysilan (APTMS) solution for 2 min and rinsed afterwards with H_2_O and Aceton.

**Transformation with 1,4-Dibrombutan.**

Samples were transferred in a mixture of 1,4-Dibrombutan, Nitromethan (CH_3_NO_2_), and Triethylamin for 2 h at 65°C, followed by rinsing with Nitromethan and air drying.

### 2.3. Coating with Poly-(4-vinylpyridinium) with or without Hexylation

In order to create a hexylated polymer-coating pretreated glass, slides were transferred in a solution consisting of poly-(4-vinylpyridinium, Sigma-Aldrich, Germany), 1-Bromhexan, and Nitromethan, incubated at 75°C overnight, rinsed thoroughly in Methanol, and finally air dried.

Procedure for control samples (standard glass slides without amorphous or nanoporous surfaces; nonhexylated polymer-coating) was comparable with the exception of missing 1-Bromhexan.

Resulting four groups of polymer-coated samples (A = nonhexylated polymer on purified glass; B = hexylated polymer on purified glass; C = hexylated polymer on amorphous silicon dioxide; D = hexylated polymer on nonoporous silicon dioxide) together with untreated glass control (E) were used in physical and biological assays to determine the influence of different surface modifications regarding biocompatibility and bactericidal effectiveness.

### 2.4. Surface Roughness—Atomic Force Microscopy (AFM) Measurements

**Figure 1:**
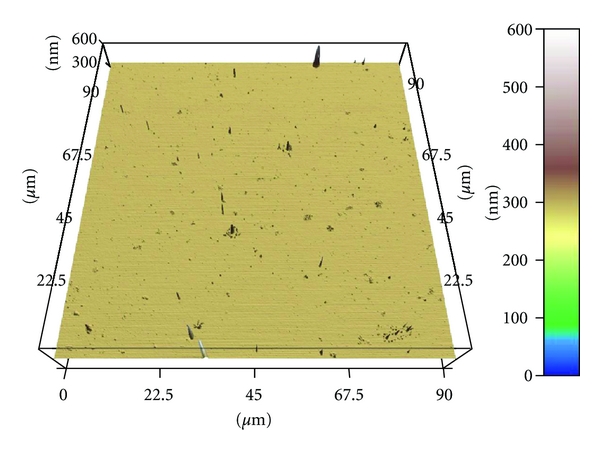
Example of an AFM illustration representing surface roughness (C = hexylated polymer spin coated with amorphous silicon dioxide).

Small-scale differences in surface roughness of polymer-coated and uncoated glass surfaces were measured by atomic force microscopy (AFM). These samples were air dried and then firmly mounted on a glass disc using double-sided adhesive tape. The surface topography of each sample was probed by contact AFM (Asylum Research MFP-3D, Santa Barbara, Calif, USA) using a standard silicon nitride tip (Olympus OMCL AC240TS). Two parameters, RMS (nm) and Average Dev (nm), characterizing surface roughness, were determined for each sample, using the IGOR Pro with the WaveMetrics data treatment software package. Values for RMS and Average Dev were calculated from the centre of the samples A–E (Figure 1).

### 2.5. Cell Culture

For the investigation of biocompatibility on polymer-coated glass, human gingiva fibroblasts of the eighth passage were used (HGFIB, Oligene, Germany). To investigate the amount of adhered cells on the different coating modifications, cell culture was performed using standard culture procedures. The fibroblasts were grown on 175 cm^2^ cell culture flasks to approximately 80% confluence in a 10% CO_2_ atmosphere at 100% humidity and 37°C. The medium used was standard Dulbeco's modified eagle medium (DMEM, Biochrom, Germany) containing 10% fetal bovine serum (FBS, PAN, Germany) and antibiotics (100 U/mL medium of penicillin, 100 *μ*g/mL streptomycin). When substantial fibroblast growth had occurred, the cells were washed with Hank's buffered salt solution (HBSS, PAA, Germany) without Ca, Mg, and phenol red, released with trypsin (Trypsin/EDTA, 0.25% of trypsin, 0.02% of EDTA). Trypsinization was stopped using culture medium, and the cells were counted and seeded into 24-well microtiter plates.

### 2.6. Standard Curve

Cells were quantified on the basis of the measured LDH activity.

24-well plates containing dilution series from human gingiva fibroblasts (10^5^, 5 × 10^4^, 2.5 × 10^4^, 1.5 × 10^4^, 5 × 10^3^, 2.5 × 10^3^ cells per well) were seeded as 4-fold samples for the standard curve. After 24 and 72 hours, two 24-well plates were used; one for the detection of the seeded cell count and the other one for the evaluation of the total lactate dehydrogenase activity per well.

### 2.7. Calculation of Cell Number

Cell counting was performed after 24 and 72 hours using an inverse microscope (Nicon Eclipse TS 100, Nikon Corporation, Tokyo, Japan). Cells in a 24-well plate were released with trypsin and calculated in a counting chamber (Neubauer) by counting 16 values per dilution.

### 2.8. Lactate Dehydrogenase Assay

**Figure 2:**
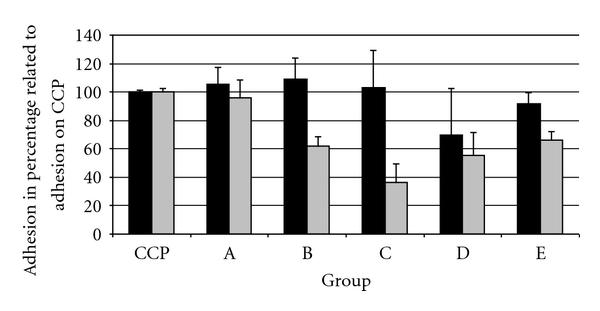
Adherent cells after 24 h (black) and 72 h (grey) of incubation relative to reference plastic (CCP = cell culture plastic; A = nonhexylated polymer; B = hexylated polymer; C = hexylated polymer spin coated with amorphous silicon dioxide; D = hexylated polymer spin coated with nanoblown silicon dioxide; E = untreated glass).

Coated glass discs from all types of polymer coatings and uncoated glass discs were extracted from the wells and rinsed with HBSS. Cells were lysed with Triton-X-100 (Sigma-Aldrich, Germany). A color reaction (Cytotoxity Detection Kit, Roche, Germany) was used to quantify the released lactate dehydrogenase at 492 nm and 650 nm with a photometer (Tecan infinite F200 Multifunctional Reader). The results of the 4 wells per seeding density were averaged. For each dilution, the total LDH release of all cells that adhered in one well was set in relation to the total cell count determined by cell counting. The standard curve was a second-order regression (Figure 2).

### 2.9. Analysis of Test Items

Samples of each group A–D, as well as the control, were placed in 24-well plates with a concentration of 1.5 × 10^4^ cells per well per mL in each well. The adherent cells on the surface were measured after 24 and 72 hours with the lactate dehydrogenase assay. The test items were washed with HBSS and transferred into new 24-well plates. That procedure avoids distortion of the results from cells that adhere to surrounding plastic.

The results were expressed as cell count per cm^2^ of the surface area, relative to the growth on cell culture plastic as 100%, and graphically illustrated.

To identify the rate of proliferation of all adhered cells, the cell count per cm^2^ of the surface area was compared between 24 and 72 hours. The resulting rates of growth were illustrated in bar diagrams.

### 2.10. Confocal Laser Scanning Microscopy

**Figure 3:**
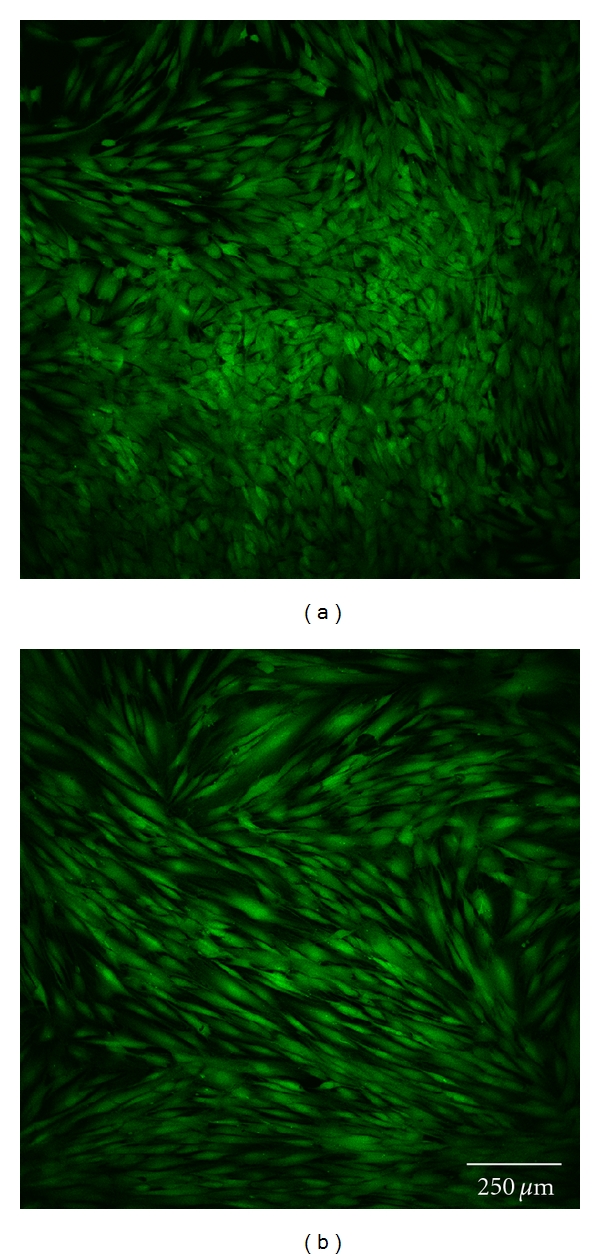
Fibroblast cells cultured on glass discs coated with nonhexylated polymer after 24 h (a) and 72 h (b).

Cell morphology was investigated by staining each sample with 5 *μ*g/mL calcein (Invitrogen, Germany). Subsequently, one uncoated and one polymer-coated glass disc with each type of surface modification were analyzed by confocal laser scanning microscopy at 24 and 72 h (Figures 3(a) and 3(b)–Figures 7(a) and 7(b)). Before staining, the cells were washed twice with HBSS, incubated for 10 minutes, and finally washed once again with HBSS before optical examination with the confocal laser scanning microscope (CLSM, Leica TCS SP5). This microscopic method gives high-resolution optical images and allows 3-dimensional reconstruction of topologically complex objects.

### 2.11. Bacterial Culture

Bacterial adhesion to pVP-coated glass sheets in comparison with the control sample was studied with *Streptococcus mutans* and *Streptococcus sanguinis* (Figures 8 and 10).

Pure cultures of bacterial strains were prepared and frozen in aliquots as stocks. For cultivation, both bacterial strains were inoculated into tryptic soy broth medium (TSB, 30 g Trypticase Soy Broth (Becton, Dickinson), 3 g yeast (Roth, Germany); pH 7.1–7.3 adjusted with 37% HCl (J.T. Baker, Holland)), grown to reach the late stationary phase, and incubated under rotation (700 rpm) for 24 hours at 37°C. Cultures were centrifuged at 2000 g and 4°C for 15 min. The bacterial pellet was washed twice in 10 mL of 50 mM Tris-HCl buffer (pH 7.5; 1 : 20 dilution with doubly distilled water of 121.14 g Tris (Roth, Germany) and 37% HCl (J.T. Baker, Holland)). Bacteria were then resuspended in 20 mL of the same buffer. Specimens coated with *Streptococcus mutans* were plated at an absorbance of 1.242 (equivalent to 6 × 10^7^ cfu/mL) and samples covered with *Streptococcus sanguinis *at an absorbance of 1.193 (equivalent to 4,7 × 10^7^ cfu/mL). The test items were incubated in a wet chamber under gentle rotation for 1 h at 37°C, then rinsed 6 times with 1 mL doubly distilled water, and fixed in 2.5% glutaraldehyde solution (Roth, Germany, 1 : 10 dilution with PBS) for 30 min at 4°C. Afterwards, bacterial microcolonies were kept cool at 4°C for 24 h, covered with 1 mL phosphate buffered saline (PBS, w/o Ca, Mg; low endotoxin; Fa. Biochrom, Germany). Microorganisms were stained with 1% acridine orange (Roth, Germany, 1 : 10 dilution with 50% EtOH) and incubated for 1 h at room temperature. Subsequently, glass sheets were rinsed with distilled water to remove excess dye, then coated with 2 mL PBS, and analyzed with the confocal laser scanning microscope at a 40-fold and 63-fold magnification.

### 2.12. Statistical Analysis

Documentation and evaluation of the data was performed with the data processing program SPSS/PC Version 18.0 for Windows (SPSS Inc., Chicago, Ill, USA). Comparison of bacterial adhesion on different polymer surfaces was performed with ANOVA test, after testing for equality of variance with the Scheffé or Tamhane tests, with a significance level of *P* ≤ 0.05 (Figures 9 and 11).

## 3. Results

### 3.1. Surface Roughness—Atomic Force Microscopy (AFM) Measurements

Polymer-coated glass tended to be rougher than untreated glass (Table 1, Figure 1).

### 3.2. Cell Culture Experiment

#### 3.2.1. Standard Curve

The relation of the measured LDH concentration and the number of fibroblasts, based on cell counts, was shown by means of a second-order polynomial regression. The coefficient of determination *R* ^2^ was 0.9983 after 24 h and 0.9961 after 72 h, which are close to the optimum value of 1.

#### 3.2.2. Lactate Dehydrogenase Assay

The counts of adhered cells after 24 h were similar in samples of groups A, B, and C. Fluctuations relative to the cell culture plastic control were less than 10%, which is within experimental inaccuracy (Figure 2). On the other hand, there was a marked reduction in the count of adhered fibroblasts in sample D.

After 72 h, the cell count for group A was approximately the same as for cell culture plastic. The cell counts for groups B and D and for purified glass were similar to each other, but lower than for group A and cell culture plastic. The cell count for group C was even lower (Figure 2).

#### 3.2.3. Proliferation Rate

To determine the proliferative behaviour of cells on different surfaces, the cell counts per cm^2^ growth surface area were compared at 24 and 72 h. The best conditions for cell proliferation were provided by cell culture plastic, followed by the controls and group A, these groups exhibited both typical morphology and high fibroblast growth. Proliferation was lower in groups B and C. The rate of proliferation was high in group D, although cell adhesion after 24 h was relatively low.

#### 3.2.4. CLSM Investigation

**Figure 4:**
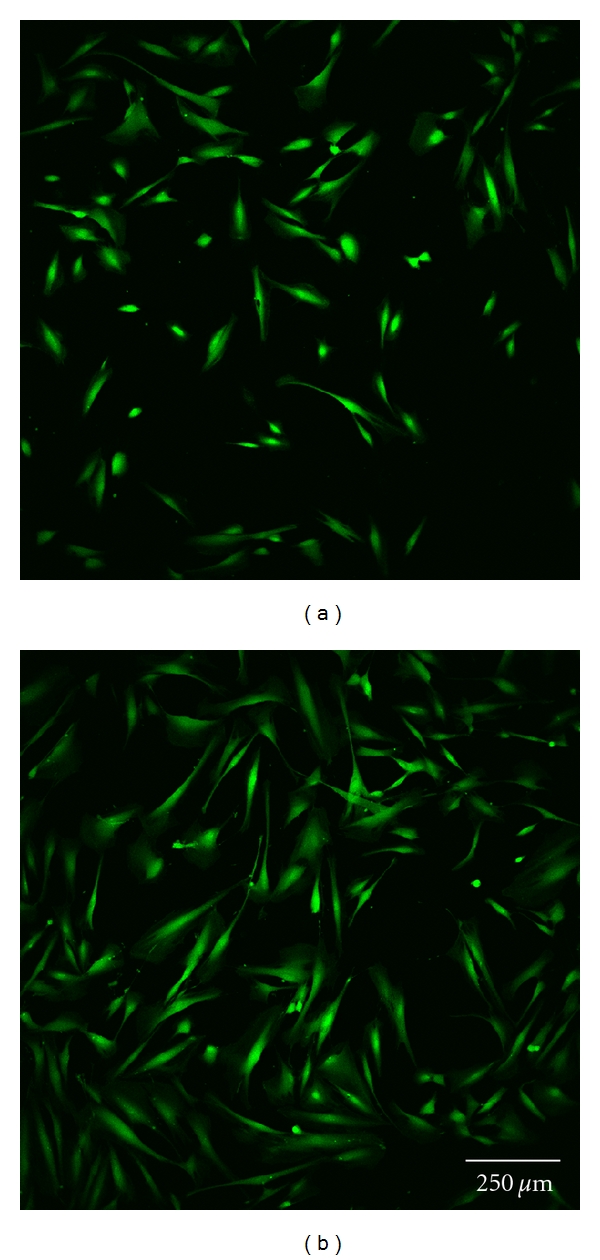
Fibroblast cells cultured on glass discs coated with hexylated polymer after 24 h (a) and 72 h (b).

**Figure 5:**
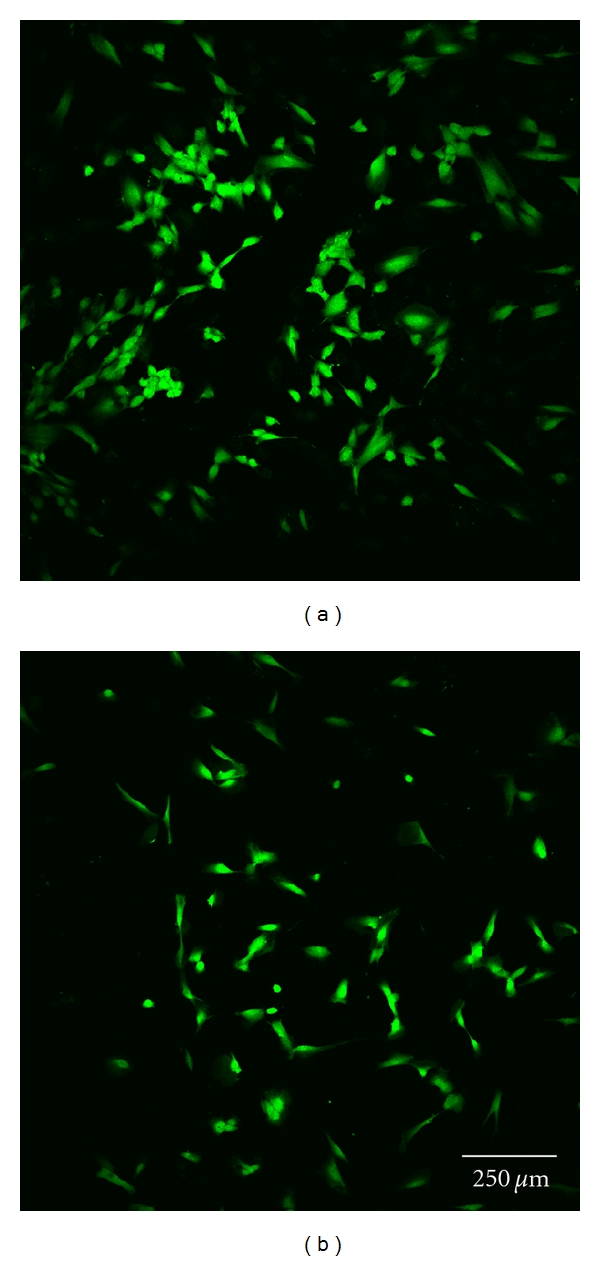
Fibroblast cells cultured on glass discs coated with hexylated polymer and spin coated with amorphous silicon dioxide after 24 h (a) and 72 h (b).

**Figure 6:**
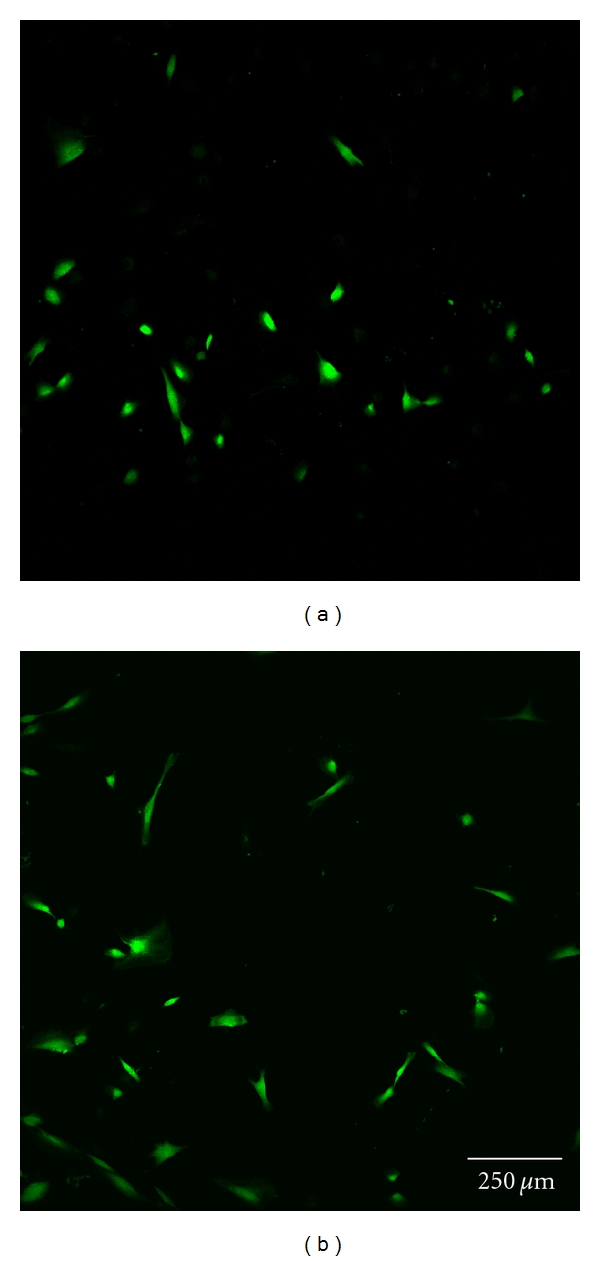
Fibroblast cells cultured on glass discs coated with hexylated polymer and spin coated with nanoporous silicon dioxide after 24 h (a) and 72 h (b).

**Figure 7:**
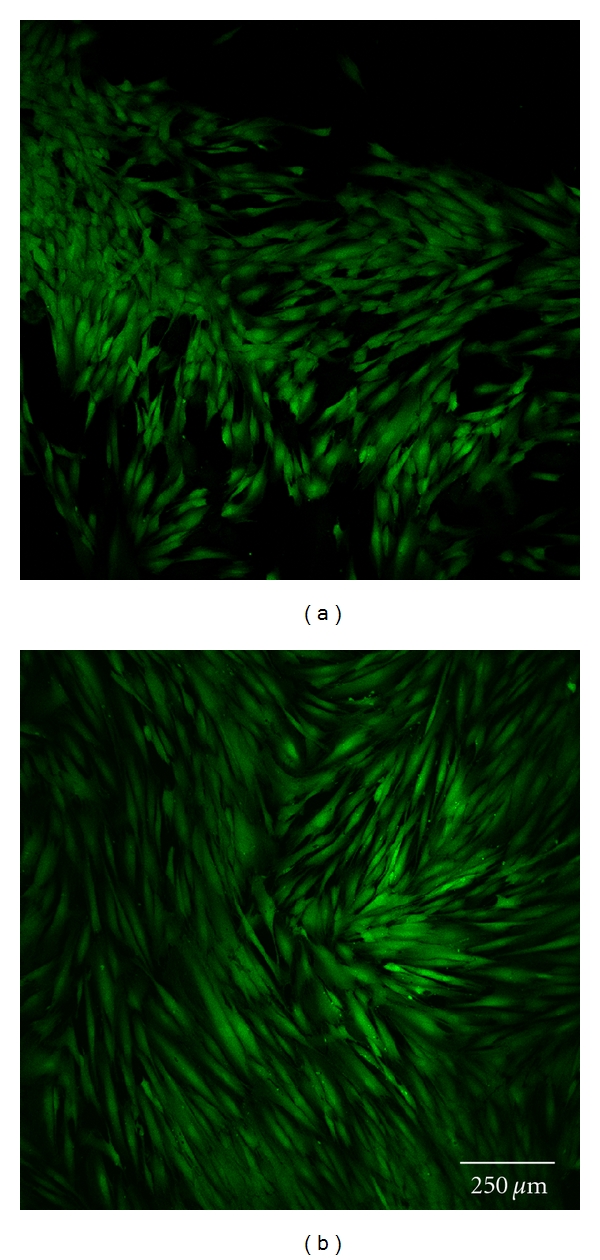
Fibroblast cells cultured on purified glass after 24 h (a) and 72 h (b).

The results of the microscopic investigations after 24 h were compared with the results of the LDH assay. The controls and group A exhibited completely adhered human gingiva fibroblasts (Figures 3(a) and 7(a)). Less cell adhesion was seen in groups B and C (Figures 4(a) and 5(a)). This might be explained if the fibroblasts are only weakly bound and are then lost during staining, transport, and storage in saline. This was consistent with the atypical morphology seen in groups B, C, and D, with very rounded cells. Cell count appeared to be lower than with purified glass and sample A. Cells might seem smaller and more compact or might have been lost during transportation and washing, which would not be seen in the LDH values as long as the intracellular LDH concentration remains unaffected. Sample D exhibited the lowest count of fibroblasts, which were incompletely attached. This is consistent with the LDH results (Figure 6(a)).

There were some differences between the LDH measurements and calcein staining after 72 h. The control, group A, and cell culture plastic all exhibited completely adhered fibroblasts (Figures 3(b) and 7(b)). The fibroblasts in groups B and C were less spread and more rounded (Figures 4(b) and 5(b)). The cell count was apparently lower on purified glass and group A, which was supported by LDH values. Group D exhibited similar morphology and counts to groups B and C (Figure 6(b)).

### 3.3. Bacterial Culture Studies

#### 3.3.1. CLSM Micrographs with *Streptococcus mutans*

**Figure 8:**
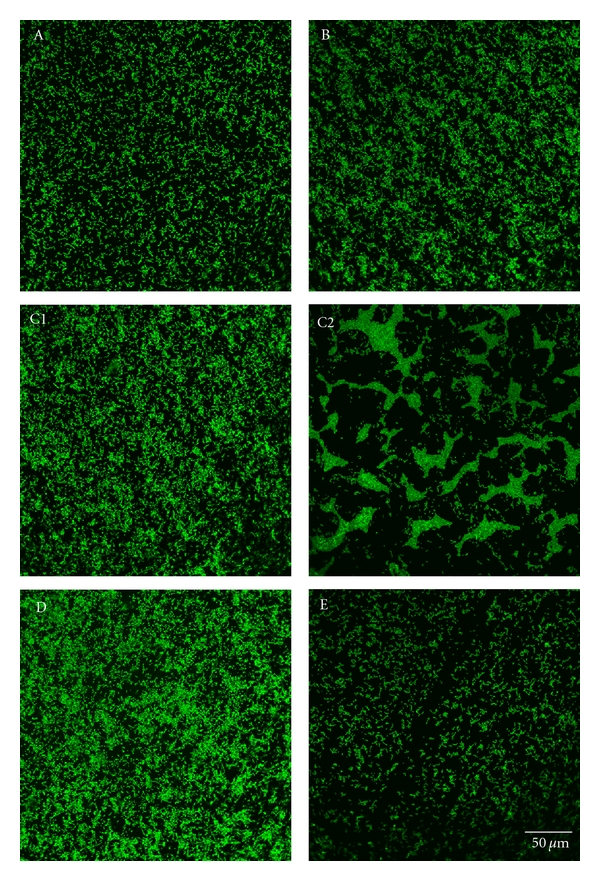
*Streptococcus mutans* cultured on glass coated with nonhexylated polymer (A), glass coated with hexylated polymer (B), glass coated with hexylated polymer and spin coated with amorphous silicon dioxide (C1 = middle; C2 = border), glass coated with hexylated polymer and spin coated with nanoporous silicon dioxide (D), and untreated glass (E).

The controls and group A exhibited short chains of *Streptococcus mutans*, with constant seeding. Controls and group A exhibited dense bacterial adhesion, which was somewhat greater in group A. The rate of seeding, cell density, and adhesion were even higher in group B, which also exhibited aggregate formation. Bacterial adhesion was lower in group C than in groups A, B, and D. In group C, seeding density was constant in the middle of the glass sheet, although there were abnormalities round the edges, with aggregate formation and less bacterial adhesion. Sample D exhibited constant seeding and the highest amount of bacterial adhesion—possibly caused by multilayer and aggregate formations (Figure 8).

**Figure 9:**
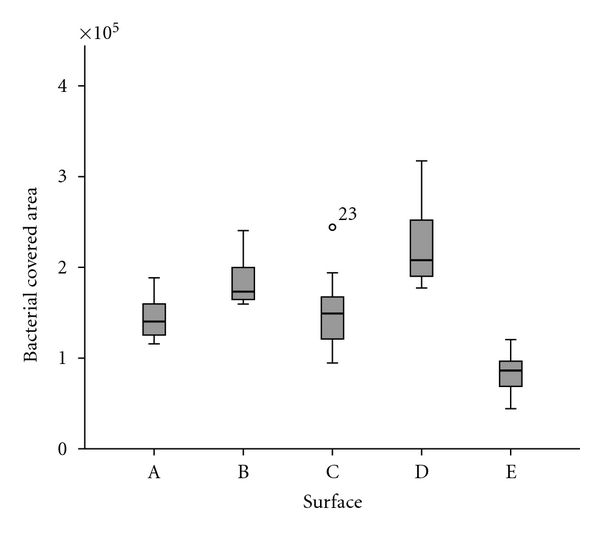
Quantification of bacterial adhesion (*Streptococcus mutans*) on glass coated with nonhexylated polymer (A), glass coated with hexylated polymer (B), glass coated with hexylated polymer and spin coated with amorphous silicon dioxide (C1 = middle; C2 = border), glass coated with hexylated polymer and spin coated with nanoporous silicon dioxide (D), and untreated glass (E); significant differences (*P* ≤ 0.05) shown between all surface modifications and untreated glass or between surface A, C in comparison to D (Tamhane).

Surface growth of *Streptococcus mutans* was quantitatively similar in groups A, B, and C. Bacterial growth was higher than this in group D, but lower on purified glass (Figure 9).

#### 3.3.2. CLSM Micrographs with *Streptococcus sanguinis*

**Figure 10:**
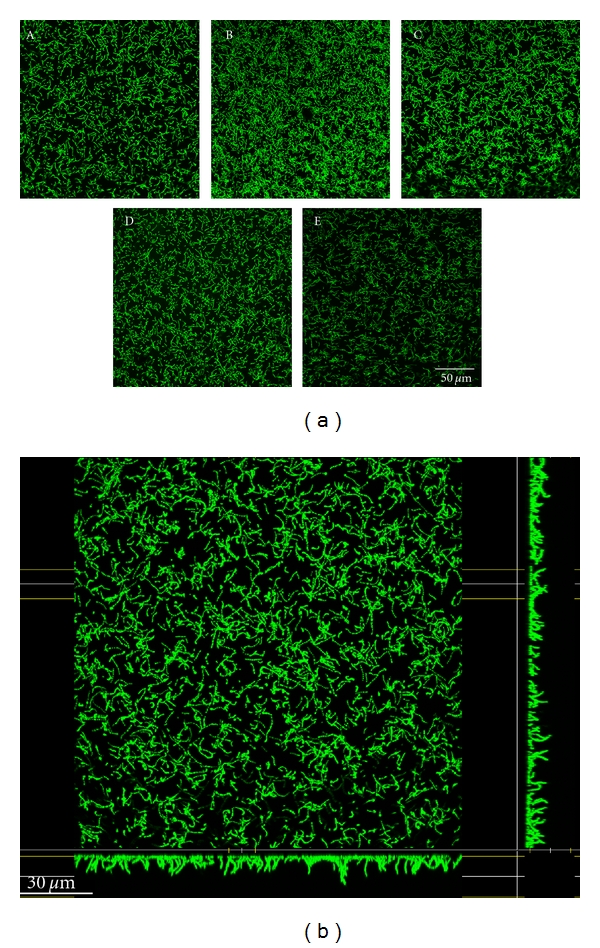
(a) *Streptococcus sanguinis* cultured on glass coated with nonhexylated polymer (A), glass coated with hexylated polymer (B), glass coated with hexylated polymer and spin coated with amorphous silicon dioxide (C), glass coated with hexylated polymer and spin coated with nanoporous silicon dioxide (D), and untreated glass (E). (b) Noticeable bacterial adhesion of *Streptococcus sanguinis *on the border of glass coated with hexylated polymer and spin coated with amorphous silicon dioxide (C).

Constant seeding and dense bacterial adhesion were consistently found with *Streptococcus sanguinis*. Groups A, B, and the control exhibited long chains of *Streptococcus sanguinis*. The lowest bacterial density was found in the control (Figure 10(a)). There were no marked quantitative differences.

Group C exposed noticeable bacterial adhesion of *Streptococcus sanguinis *on the border of the surface where bacterial chains are standing vertically to the surface (Figure 10(b)).

**Figure 11:**
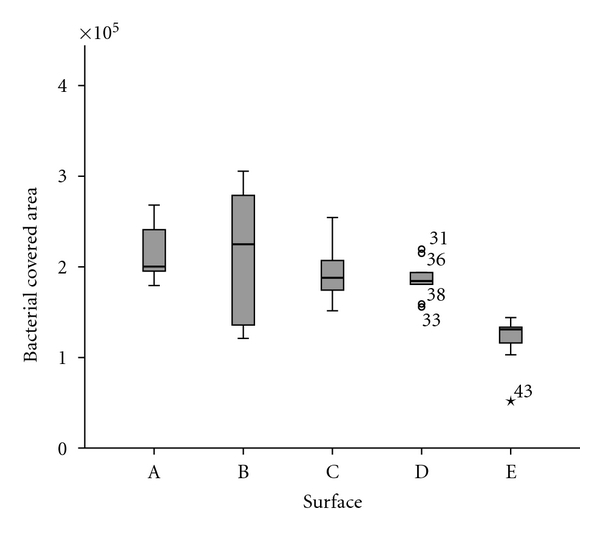
Quantification of bacterial adhesion (*Streptococcus sanguinis*) on glass coated with nonhexylated polymer (A), glass coated with hexylated polymer (B), glass coated with hexylated polymer and spin coated with amorphous silicon dioxide (C1 = middle; C2 = border), glass coated with hexylated polymer and spin coated with nanoporous silicon dioxide (D), and untreated glass (E); significant differences (*P* ≤ 0.05) found between all surface modifications and untreated glass (Scheffé test).

Bacterial adhesion was lowest with purified glass (Figure 11).

## 4. Discussion

The long-term success of osseointegrated dental implants is based on a complex interaction of different factors [18, 20, 21]. Among them, constant bacterial biofilm formation as well as an inefficient adhesion of soft tissue belong to the most serious and momentary-deficient-solved problems which abet peri-implantitis and may lead to early oral implant failure [3, 12–14, 22]. Therefore, in the present study not only the promising antibacterial qualities of pVP-coatings were analysed concerning typical oral bacteria but also the cytotoxicity of differently coated glass slides as well as the effects of surface preconditioning in terms of surface enhancement.

Because of esthetical reasons as tooth-like translucence as well as advantages in biocompatibility, low plaque adhesion, fracture toughness, combined with a suitable use in veneering metal framework such as implants, the standing of dental ceramics will increase in future [20, 23, 24]. Since glass and ceramics both provide silicate groups on their surfaces, which are adaptable to mediate polymer binding, glass discs were used in the experiments as a model substrate coated with pVP. Same is the case for surfaces made of amorphous and nanoporous silicon dioxide, but differences exist in the surface structure, as silicon dioxide has a larger surface than purified glass. In addition, nanoporous silicon dioxide has pores in the nanometer range, which might qualify a nanoporous coating to amplify the surface area and to insert agents into the pores for chemical modifications.

In contrast to the results of Tiller et al. within the present study, oral bacteria like *S. mutans* and *S. sanguinis* showed a significantly increased adherence (*P* ≤ 0.05) on all polymer-coated samples in comparison to the untreated glass control. Thereby, it seemed less relevant for bacterial adhesion whether the pVP was hexylated or the substrate was preconditioned with any kind of silicon dioxide. *In vitro* studies have indicated that the chemical compositions, roughness, hydrophobic properties, and charges of biomaterial surfaces have a strong influence on microbial adhesion and subsequent biofilm formation [6, 12, 25–27]. As the polymer-coated samples turned out to be rougher than untreated glass, this might tend to increase bacterial adhesion although in another study only surface roughness above 0.2 *μ*m significantly impacted plaque formation [13]. In the present study, the control glasses and all polymer treatments lie well below these limits. Nevertheless, differences in roughness of surface modifications were observed which corresponded with the density of bacterial adhesion. The surface charge of used samples was already characterized by means of contact angle measurement and a fluorescein staining test to control the connection between the polymers to purified glass modified in various ways [19]. Contact angle measurement revealed a greater surface charge of nonhexylated pVP than of purified glass, whereas hexylated pVP exhibited the highest. An increasing contact angle indicates an enhancement of hydrophobicity [19, 28], which correlates with advanced microbial adhesion depending on the combination of organism, medium, and substratum [28, 29]. Regarding the fluorescein staining test, this dye binds to quaternary amino groups present after polymer hexylation. In case of hexylated pVP, the density of charged amino groups conducted 4.5 and 4.9 *μ*mol ∗ L^−1^ ∗ cm^−2^, whereas for example, octylated and decylated polymers featured lower concentrations, resulting in less antibacterial activity [19]. Thereby, the coating with hexylated polymer correlates with a higher concentration of quaternary amino groups indicating an improved and more effective binding of the polymer onto the surface and leads to increased hydrophobicity and more attraction for bacteria at the same time [16, 19, 30, 31].

Another important aspect in coating effectiveness of course might be seen in surface thickness, which can be influenced amongst others by pretreatment of the substrate [32–34]. Investigations with ellipsometry by Wagner [35] indicated significant differences between the noticeable amorphous coating (50 nm) and the hexylated surface without silicon dioxide (5 nm). The experimental results of the present study revealed a special effect on the amorphous coating, which may associate to this point. Adhered *S. mutans* showed massive aggregates and less bacterial adhesion only at the border of the test items (Figure 8), indicating a bacterial stress reaction to an insufficient surface, whereas chains of *S. sanguinis* appeared partially erected and standing vertically to the base likewise avoiding surface contact (Figure 10(b)). In consequence, the nonuniform disposition of polymer between centre and border of samples by spin coating together with the magnified surface of amorphous silicon dioxide overcame in some areas just the critical amount, which is required for an antibacterial effect. Actually, this correlates with the observation of Tiller et al. in a continuative study with pVP describing a bactericidal activity by increasing the surface density of pyridinium groups [36]. Also, Kenawy et al. reflected that the growth of gram-negative and gram-positive bacteria depended on a certain polymer structure, formation of the active group, and decreased with a higher polymer concentration [37].

Finally, the different physiology of bacteria could be responsible for the differing results in comparison to Tiller et al. As the antibacterial activity of polymers was described more effective against gram-negative than gram-positive bacteria in other studies [37], the chosen gram-positive facultative anaerobic microorganisms, which represent typical oral bacteria, might be less sensitive to the analysed polymer-coating.

Since Tiller et al. did not consider the effects of pVP coating on tissue-implant connection, the other major aspect of the present study was the examination of gingival fibroblast adhesion and proliferation on polymer-coated versus uncoated substrates. Regarding initial adhesion after 24 h only nanoporous silicon dioxide revealed some reduction in the quantification assay. In contrary, additional fluorescence pictures raise questions about the quality of this adhesion in case of all samples coated with hexylated pVP, because of more rounded instead of fibroblastoid spreaded cells. Likewise, after 72 h, amount and morphology of adhered cells were best for the controls and glass with nonhexylated polymer coating, whereas surfaces with hexylated pVP lag behind, also demonstrated by a decreased or even negative proliferation rate, possibly as a consequence of poor morphology and cell adhesion. Summarized the results of the study showed that the potentially bactericidal polymer poly-(4-vinyl-N-hexylpyridiniumbromide) itself has no cytotoxic effect on human gingival fibroblasts and, therefore, could be used as an origin for future coating strategies regarding dental implants. However, necessary hexylation of polymer with view to antibacterial properties comes along with a loss of biocompatibility. So a combination with other polymers and a creation of copolymer coatings for glass might be a reasonable way to improve cellular adhesion and proliferation as showed already for titanium substrate by a study of Heuer et al. [18]. In addition, Ortega et al. described the insertion of a copolymer that might provide the formation of hyperbranched polymers containing terminal ammonium groups as antimicrobial agents that might enhance a gradual delivery of biocides [38].

## 5. Conclusion

A favourable implant surface is characterized by reduced bacterial adhesion with simultaneous good biocompatibility. The results of the present study revealed no cytotoxic effect of poly-(4-vinyl-N-hexylpyridiniumbromide) on human gingival fibroblasts, that might indicate the polymer as a prospective coating for the application on oral implant surfaces although the effects of hexylation have to be discussed critically. However, the findings of the known antibacterial polymer could not be confirmed within our study because no bactericidal activity on differently coated glass slides regarding typical oral bacteria could be accomplished. An improved surface roughness, thickness, alkylation, and configuration of the polymer in regard of continuative use of oral bacteria ought to be considered in further investigations.

## Figures and Tables

**Table 1:** Mean measurements of surface roughness estimated by AFM, Random Mean Square (RMS), and Average Deviation (Average Dev); glass coated with nonhexylated polymer (A), glass coated with hexylated polymer (B), glass coated with hexylated polymer and spin coated with amorphous silicon dioxide (C), glass coated with hexylated polymer and spin coated with nanoporous silicon dioxide (D), and untreated glass (E).

		A	B	C	D	E
RMS	[nm]	1.379	6.558	5.078	5.793	1.176
Average Dev	[nm]	0.515	1.233	0.611	4.279	0.906
